# Different Diversity and Distribution of Archaeal Community in the Aqueous and Oil Phases of Production Fluid From High-Temperature Petroleum Reservoirs

**DOI:** 10.3389/fmicb.2018.00841

**Published:** 2018-04-27

**Authors:** Bo Liang, Kai Zhang, Li-Ying Wang, Jin-Feng Liu, Shi-Zhong Yang, Ji-Dong Gu, Bo-Zhong Mu

**Affiliations:** ^1^State Key Laboratory of Bioreactor Engineering and Institute of Applied Chemistry, East China University of Science and Technology, Shanghai, China; ^2^Shanghai Collaborative Innovation Center for Biomanufacturing Technology, East China University of Science and Technology, Shanghai, China; ^3^School of Biological Sciences, The University of Hong Kong, Pokfulam, Hong Kong

**Keywords:** aqueous phase, oil phase, archaeal community, petroleum reservoir, water flooding, indigenous microorganisms, exogenous microorganisms

## Abstract

To get a better knowledge on how archaeal communities differ between the oil and aqueous phases and whether environmental factors promote substantial differences on microbial distributions among production wells, we analyzed archaeal communities in oil and aqueous phases from four high-temperature petroleum reservoirs (55–65°C) by using 16S rRNA gene based 454 pyrosequencing. Obvious dissimilarity of the archaeal composition between aqueous and oil phases in each independent production wells was observed, especially in production wells with higher water cut, and diversity in the oil phase was much higher than that in the corresponding aqueous phase. Statistical analysis further showed that archaeal communities in oil phases from different petroleum reservoirs tended to be more similar, but those in aqueous phases were the opposite. In the high-temperature ecosystems, temperature as an environmental factor could have significantly affected archaeal distribution, and archaeal diversity raised with the increase of temperature (*p* < 0.05). Our results suggest that to get a comprehensive understanding of petroleum reservoirs microbial information both in aqueous and oil phases should be taken into consideration. The microscopic habitats of oil phase, technically the dispersed minuscule water droplets in the oil could be a better habitat that containing the indigenous microorganisms.

## Introduction

Subsurface petroleum reservoir ecosystems are complex and generally considered extreme conditions, including hypoxic state, high temperature, pressure, salinity, and hydrophobicity (Li et al., 2007). It harbors wide distribution of anaerobic microorganisms including sulfate-reducing bacteria, nitrate-reducing bacteria, fermentative bacteria, syntrophic bacteria, methanogens, and many more microorganisms (Orphan et al., 2001; Grabowski et al., 2005; Kleikemper et al., 2005; Youssef et al., 2009; Li et al., 2011; Mayumi et al., 2011; Meckenstock et al., 2016). A review of microbial communities in natural oil reservoirs and associated environments showed that comparing to bacteria, archaea distribution in petroleum reservoir is less diverse (Li et al., 2017), but performing fundamental and even indispensable functions, such as hydrocarbon degradation (Khelifi et al., 2014; Mardanov et al., 2015), ammonia oxidation (Li et al., 2011), sulfate reduction (Stetter et al., 1987), and methanogenesis (Kleikemper et al., 2005), etc. However, some limitations like uncultivated status, comparatively low abundance, and limited database all restricted the further research of their phylogenetic diversity, metabolic capability, and ecological roles (Yan et al., 2018). Particularly for part of unclassified archaeal members, their potential new capability of anaerobic hydrocarbon degradation cannot be ignored (Mardanov et al., 2009; Khelifi et al., 2014). Thus, a better knowledge of the archaea ecology in petroleum reservoirs is of great importance (Head et al., 2003; Aitken et al., 2004).

The environmental factors (physical and chemical characteristics) of an ecosystem may vary greatly across different subsurface petroleum reservoirs and are thought to determine and influence the living organisms to survive or thrive (Magot et al., 2000; Sierra-Garcia et al., 2017). The free-living microorganisms exhibit non-random distribution patterns across diverse habitats (Ge et al., 2008; Stegen et al., 2012; Hatosy et al., 2013; Kuang et al., 2013; Lin et al., 2013; Briggs et al., 2014; Liang et al., 2015). Temperature, salinity, pH, and nutrient appear to be the major environmental factors that can play important roles for the types of microorganisms and metabolic activities in oil field environments (Magot et al., 2000; Youssef et al., 2009; Piceno et al., 2014; Adelaja et al., 2015). Among of which, temperature has been considered the highest theoretical limiting factor controlling microbial growth in petroleum reservoirs (Magot et al., 2000).

Because of the difficulties of sampling and extracting microorganisms directly from oil phases of the deep subsurface ecosystems, previous practices have been mainly focused on collecting microorganisms from production fluid to analyze the subsurface microbial community. Recently, efforts were made to characterize microorganisms collected both in separate aqueous phase and oil phase from production fluid samples and results showed that microorganisms in the subterranean oil phases were different from those in the aqueous phases (Kobayashi et al., 2012; Kryachko et al., 2012; Wang et al., 2014; Cai et al., 2015). It is clear that further investigations on microbial communities in aqueous and oil phases of petroleum reservoirs will allow a better understanding on the microbial community, biochemical processes, and also the functioning microorganisms under original or long-term water flooding subsurface environments.

To narrow the gap in our knowledge of how archaeal communities can differ between oil and aqueous phase and whether environmental factors promote substantial differences in microbial communities among production wells, we studied the taxonomic diversity of archaeal communities in both aqueous and oil phases from four production wells in the high-temperature petroleum reservoir. Bacterial distribution pattern has been published previously (Wang et al., 2014); in this paper, we focus on the composition and distribution of archaea that vary with temperature, water flooding, oil, and aqueous phases, etc. 16S rRNA gene-based molecular analysis was used to investigate the archaeal community structures by 454 high through pyrosequencing.

## Materials and Methods

### Sample Collection

Samples were collected from Chengdong petroleum reservoir in Shengli oilfield, located in Yellow River Delta of Shandong province adjacent to the Bohai Bay of China. Chengdong petroleum reservoir has been water flooded by the oil company for >30 years, with low oil recovery and high water cut. Four production wells with temperature ranged from 55 to 65°C and different water contents (C7–J9 with 98.0%, C6–15 with 93.8%, C6–G10 78.6%, and CN13–13 with 66.8%) were selected for the detection of archaea diversity and distribution. Eight samples were obtained from aqueous and oil phases of the four production wells (C7–J9, C6–15, C6–G10, and CN13–13) of Shengli oil field and were named as A1-Aqueous, A2-Aqueous, A3-Aqueous, A4-Aqueous, A1-Oil, A2-Oil, A3-Oil, and A4-Oil, respectively. Detailed characteristics of the four production wells were provided by the local oil company Sinopec (the China Petroleum & Chemical Corporation) and shown in **Table 1**.

**Table 1:** Characterization of the four production wells with different water cut from petroleum reservoir (nd, not detected).

	A1 (C7–J9)	A2 (C6–15)	A3 (C6–G10)	A4 (CN13–13)
Water cut (%)	98.0	93.8	78.6	66.8
Cl^-^ (mg L^-1^)	3338.3	3480.8	3205.7	3782.2
${\mathrm{SO}}_{4}^{2–}$ (mg L^-1^)	12.7	Nd	180.2	19.9
K^+^ and Na^+^ (mg L^-1^)	2365.2	2521.0	2362.6	2521.7
Ca^2+^ (mg L^-1^)	84.8	67.4	59.1	66.5
Mg^2+^ (mg L^-1^)	16.1	20.6	22.8	25.2
pH	8.0	8.0	7.0	7.0
Oil viscosity (mPa s)	278.0	230.8	1872.0	2866.0
Temperature (°C)	58	59	55	65
Mineralization (mg L^-1^)	6671.5	7098.3	6648.8	6899.0

Samples were collected from the wellheads of each production well, where the oil and water mixture fluid were pumped out. Mixture fluid from wellheads of four production wells was collected directly into independent clean and sterilized 5 L glass bottles till the bottles were filled up to exclude oxygen. Bottles were sealed with butyl rubber stopper to prevent intrusion of oxygen and transported back to laboratory immediately, stored at 4°C for further analysis.

### DNA Extraction

For a better separation of oil and water from the mixture fluid, mixture samples were preheated at 50°C and then were separated into aqueous phase and oil phase by using a 2 L separatory funnel. Aqueous and oil phases were collected separately for subsequent total microbial genomic DNA extraction. For aqueous phase, microbial cells were obtained after filtering through 0.22-μm-pore-size polycarbonate membranes (25 mm diameter; Millipore, Bedford, MA, United States). The polycarbonate membranes with the collected microbial cells were cut into small pieces by using the sterile scissor and placed into the sterile centrifuge tubes for cell disruption (AxyPrep^TM^ Bacterial Genomic DNA Maxiprep Kit, Axygen Biosciences, United States) following the procedures in accordance with the manufacturer’s instructions. For oil phase, three volumes of isooctane (2,2,4-trimethylpentane) were added, mixed thoroughly, and let it stand overnight at room temperature. The precipitates were obtained after centrifuged at 5000 × *g* for 30 min at 4°C, washed, and re-suspended twice with three volumes of isooctane, then centrifuged at 5000 × *g* for 30 min at 4°C. The precipitates were dried in vacuum oven at 55°C for 2 h. The supernatant fluid from this procedure was filtrated through 0.22-μm-pore-size polycarbonate membranes as described above to collect the microbial cells. Both the polycarbonate membranes with microbial cells and precipitates were collected for the total microbial genomic DNA extraction by using the AxyPrep^TM^ Bacterial Genomic DNA Maxiprep Kit (Axygen Biosciences, United States). SmartSpec Plus (Bio-Rad, Hercules, CA, United States) was used for determination of DNA concentration and electrophoresis on a 0.8% (*w*/*v*) agarose gel for confirmation. To obtain enough quantity of DNA we repeated this procedure at least three times, and pooled all the DNA from each of the eight samples (oil and aqueous phases), respectively, stored at -20°C for PCR amplification.

### PCR Amplification and 454 Pyrosequencing

For each sample, archaeal 16S rRNA gene was amplified using archaea-specific primer set 344-F (5′-ACG GGG YGC AGC AGG CGC GA-3′) and 915-R (5′-GTG CTC CCC CGC CAA TTC CT-3′) (Casamayor et al., 2002). Each sample used a specific 344-F primer with different barcode to enable the following multiplex sequencing PCR reactions were carried out in a 20-μL volume mixture containing a final concentration of 250 mM of each dNTP, 5 μM of each primer, 0.4 μL FastPfu polymerase, 2 μL of DNA template (10 ng), and de-ionized ultrapure water to make up the volume. PCR amplification program contained a 2-min initial denaturation at 95°C, followed by 25 cycles of denaturing at 95°C for 30 s, annealing at 57°C for 30 s, extension at 72°C for 30 s, with a final extension at 72°C for 5 min. PCR products were purified using a DNA purification kit (U-gene, Anhui, China) for the pyrosequencing. All the purified PCR amplicons were pooled together and send to Majorbio Bio-pharm Biotechnology Co., Ltd. (Shanghai, China) for pyrosequencing through a Roche Genome Sequencer FLX Titanium 454 Pyrosequencer (Roche, Nutley, NJ, United States). 454 pyrosequencing data were deposited into NCBI SRA database under the accession number SRA096386.

### Sequence Analysis

Raw sequence data were processed for quality control mainly using the microbial ecology community software program mothur software (Schloss et al., 2009). The valid reads were selected according to the following standard quality controls: raw sequences that did not match primers and used barcode sequences perfectly (more than two mismatches), with too short lengths (<200 bp), with ambiguous bases, and with poor quality (read quality scores <25) were excluded. Sequences were grouped by each sample based on the same barcode. Clean reads were processed for chimeras screening and removing using UCHIME (Edgar, 2010) following by the removing of barcode and primes.

Valid reads were then analyzed using Quantitative Insights into Microbial Ecology (QIIME) pipeline, version 1.9.1 (Caporaso et al., 2010). Operational taxonomic units (OTUs) were generated at 97% similarity by UCLUST using the workflow of pick_open_reference_otus.py in QIIME, and OTUs with only one read (Singletons) in the entire data set were removed. To compute alpha- and beta-diversity for all the eight samples at the same level of sampling effort (subsampling), each dataset was rarefied to 3300 reads based on the minimal sequencing depth among the whole samples. QIIME’s multiple_rarefactions.py and alpha_diversity.py beta_diversity.py were used for generating rarefied OTU tables and alpha- and beta-diversity results, respectively. Taxonomy of all 16S rRNA sequences was classified through the Ribosomal Database Project (RDP) (Wang et al., 2007) at the genus level.

### Statistical Analysis

Linear correlation between observed OTUs and temperate was determined using Pearson correlation analysis. It was also used for computing the correlation of archaeal taxa (at genus level) and environment factors, taxa with <0.01% relative abundance were eliminated.

Boxplot measured by Student’s *t*-test showing Choa1 between oil and aqueous samples. Significant effect of each environment factor was measured using PerMANOVA analyses (Adonis function, 999 permutations). Bray–Curtis dissimilarity between each sample pair was calculated using the VEGAN 2.4-6 package in R (R Core Team, 2017). Venn diagrams showing the shared OTUs (clustered under the minimal sequencing depth) in the four aqueous samples and four oil samples were generated by using the R package VennDiagram. STAMP software package (Parks and Beiko, 2010) was used for the construction of principal component analysis (PCA) of all the oil and aqueous samples. A log-normalized heatmap showing taxonomic diversity and hierarchical clustering of eight samples was performed using STAMP software package with method of average neighbor (UPGMA) and 0.75 dendrogram clustering threshold.

## Results

### Geochemical Characteristics of Production Wells

Four production wells of Shengli oil field: C7–J9 (A1), C6–15 (A2), C6–G10 (A3), and CN13–13 (A4) were the sites where we sampled for the production fluids. After over 30 years water flooding, they contained high water contents: 98.0, 93.8, 78.6, and 66.8%. Oil viscosity value was consistent with the water cut that production wells with lower water cut (A3 and A4) got much higher oil viscosity than that with higher water cut (A1 and A2). The depth ranged from 1127.9 to 1287.4 m and temperature ranged from 55 to 65°C. Comparing to other elements, concentration of Cl^-^, K^+^, and Na^+^ in the production wells is quite high, the minimum of which were 3205.7 and 2362.6 mg L^-1^. The concentration of ${\mathrm{SO}}_{4}^{2–}$ in A3 production well is much higher (180.2 mg L^-1^) than there other production wells. Detailed characteristics were shown in **Table 1**.

### Archaeal Community Structure and Composition

**FIGURE 1:**
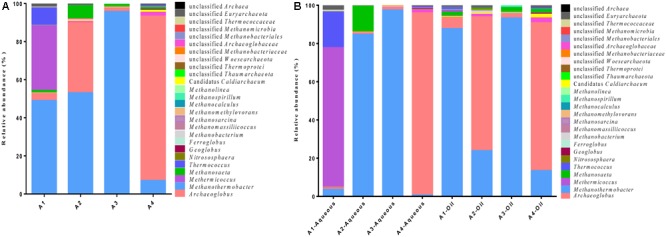
Taxonomic distribution of the archaeal communities in the production fluid samples from four production wells. **(A)** A total distribution and relative abundance of archaeal community in the four production wells (A1, A2, A3, and A4). **(B)** Relative abundance of archaeal community in the aqueous and oil phases from each four production wells.

The eight samples in aqueous and oil phases of different production wells resulted in different numbers of OTUs and OTU abundance. All the sequences classified through the RDP revealed the presence of at least 16 archaeal genera within 5 phyla (**Figure 1**). In general, *Archaeoglobus* and *Methanothermobacter* were the two most abundant division, comprising approximately up to 95.3 and 97.4% in A4-Aqueous and A3-Aqueous, respectively (**Figure 1B**). *Methermicoccus* appeared as the dominant division accounted for 72.8% in A1-Aqueous sample. Microbes in the genus of *Thermococcus* (A1-Aqueous, 18.7%) and *Methanosaeta* (A2-Aqueous, 13.4%) also had a relative high abundance. In contrast, members in the genus of *Candidatus* Caldiarchaeum (0–1.8%), *Nitrososphaera* (0–0.2%), *Geoglobus* (0–0.5%), *Ferroglobus* (0–0.03%), *Methanobacterium* (0–1.3%), *Methanomassiliicoccus* (0–0.5%), *Methanosarcina* (0–0.03%), *Methanomethylovorans* (0–0.1%), *Methanospirillum* (0–0.9%), *Methanocalculus* (0–0.1%), and *Methanolinea* (0–0.1%) represented a much smaller proportion. The remaining sequencing reads that could not be classified also occupied as the minority, the highest proportion of them was no >2.6%.

After combining the sequencing reads from aqueous and oil phases together, an overall archaeal community diversity in the four production wells with different water cut from petroleum reservoir was obtained (**Figure 1A**). *Methanothermobacter* were the most abundant division in A1 (49.0%), A2 (53.0%), and A3 (95.7%) production wells, but *Archaeoglobus* constituted the highest proportion (86.3%) *with Methanothermobacter* accounting for only 7.0% in A4 production well. Members in the genus of *Methermicoccus* and *Archaeoglobus* represented the second largest proportion in A1 and A2 at 34.0 and 36.7%, respectively.

### Archaeal Diversity and Distribution in Aqueous and Oil Phases

Eight samples of both aqueous and oil phases from four production wells were used for research in this study. Raw sequence data of archaeal 16S rRNA gene were obtained through 454 pyrosequencing. After filtering out raw sequences with low quality through quality control and chimera detection, a total of 28,704 reads were obtained. Singleton OTUs generated at 97% similarity were removed for downstream alpha- and beta-diversity analysis. Rarefaction curves of Chao1 and observed OTUs for the eight samples were shown (Supplementary Figure S1). Both the Chao1 and observed OTUs showing that A4-Oil and A3-Aqueous contained the highest and lowest archaeal diversity, respectively. Moreover, for each production well, Chao1 and observed OTUs in oil phase were much higher than that in the aqueous phase.

**FIGURE 2:**
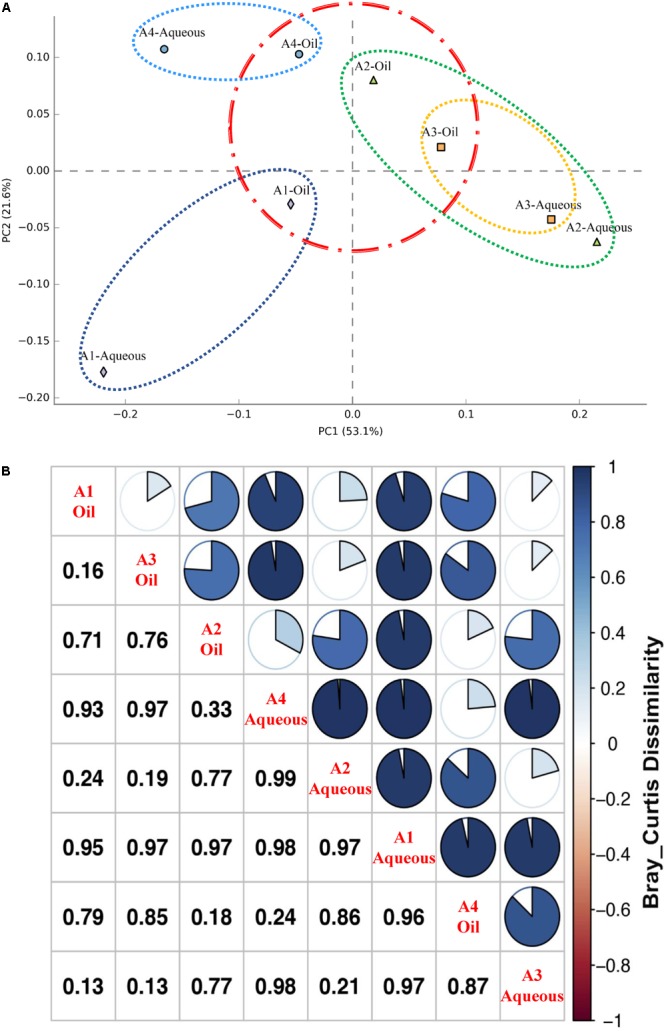
Difference of archaeal community structure for the eight samples from oil and aqueous of four production wells. **(A)** Principal component analysis (PCA) performed with archaeal phylogenetic diversity. Spots show the archaeal communities of the eight samples, filled rhombus, triangle, square, and circle representing aqueous and oil phases in A1, A2, A3, and A4 production wells, respectively. **(B)** Bray–Curtis dissimilarity showing quantity of the compositional dissimilarity between each sample pair.

To further examine possible differences in the occurrence of archaeal phenotypes from each sample, including the rare division, the 454 pyrosequencing data were further visualized using hierarchically clustered heatmap analysis on the genus level (Supplementary Figure S2). The log-normalized heatmap generally indicated two groups, A1-Aqueous sample was separated from other seven samples. In the subgroup, four oil phase samples were clustered together. A2-Aqueous and A3-Aqueous were clustered into a subgroup and separated from A4-Aqueous which was much closer to oil phase samples. These results were consistent with the PCA (**Figure 2A**). Comparing to the oil phase, archaeal communities among the samples of aqueous phase had a relative higher difference. In addition, the dissimilarity of the archaeal communities between the aqueous and oil phases from each production well was also observed, especially for those with higher water cut (A1-Aqueous/A1-Oil and A2-Aqueous/A2-Oil). Moreover, statistical analysis of Bray–Curtis dissimilarity (**Figure 2B**) showed that the numerical distance between oil and aqueous phase from A1, A2, A3, and A4 production wells were 0.95, 0.77, 0.13, and 0.24, respectively. Specifically, dissimilarity of production wells with high water cut (A1 = 98.0% and A2 = 93.8%) was much higher than those with low water cut (A3 = 78.6% and A4 = 66.8%). To better illustrate the difference of archaeal communities between oil and aqueous phases, Choa1 boxplots measured by Student’s *t*-test was drawn and *p*-value was 0.067 which was slightly more than 0.05 (Supplementary Figure S3).

Shared communities in aqueous and oil phases of the same well were further determined via the Venn diagram (Supplementary Figure S4). OTUs were cluster at 97% similarity under the minimal sequencing depth. OTU number in the aqueous and oil phases of A1, A2, A3, and A4 production wells was 121/141, 86/174, 54/74, and 139/189, respectively. For all the four aqueous samples, they shared only three OTUs while for oil samples they shared 17 OTUs. To be more specific, the shared OTUs in aqueous samples belong to *Methanothermobacter* and *Archaeoglobus*, shared OTUs in oil samples belong to *Archaeoglobus, Methanothermobacter, Methanosaeta*, and unclassified *Euryarchaeota*.

### Statistical Analysis of Correlation Between Environmental Factors and Archaeal Taxa

**FIGURE 3:**
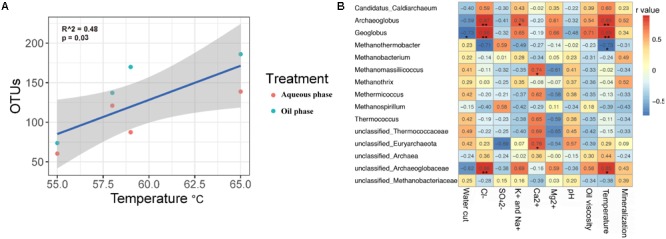
Pearson correlation analysis of environmental factors. **(A)** The linear correlation between observed OTUs and temperate (*R*^2^ = 0.48, *p* = 0.03). Observed OTUs were rarefied at the same sequencing depth (3300 reads). Blue and orange points represent samples from oil phase and aqueous phase, respectively. **(B)** The correlation between environmental variables and archaeal taxa (at genus level). Taxa with relative abundance <0.01% were eliminated. Data with one asterisk means *p* < 0.05, with two asterisk means *p* < 0.01. Color with red shows positive correlation while blue shows negative correlation. The bigger *r*-value shows the more correlative they are.

The four production wells of Chengdong petroleum reservoir showed some environment factors (**Table 1**). PerMANOVA analyses calculated the effect of environmental factors on the archaeal composition (Supplementary Table S1). It illustrated that Cl^-^ and temperature (*p* < 0.05) were the two factors that significantly affected archaeal distribution in the production wells. Linear correlation between observed OTUs and temperate was performed according to Pearson correlation analysis (*R*^2^ = 0.48, *p* = 0.03) (**Figure 3A**). Archaeal diversity in production wells was positively correlated with temperature (55–65°C).

Pearson correlation analysis showed the correlation between environmental factors and archaeal taxa (at genus level), taxa with relative abundance <0.01% were eliminated (**Figure 3B**). *Geoglobus* was negatively correlated with the water cut (*p* < 0.05). *Archaeoglobus, Geoglobus*, and unclassified *Archaeoglobaceae* were positively correlated with Cl^-^ concentration (*p* < 0.01). *Archaeoglobus* was positively correlated with K^+^ and Na^+^ concentration (*p* < 0.05). *Methanomassiliicoccus* and unclassified *Euryarchaeota* were positively correlated with Ca^2+^ concentration (*p* < 0.05). *Archaeoglobus* (*p* < 0.01), *Geoglobus* (*p* < 0.01), and unclassified *Archaeoglobaceae* (*p* < 0.05) were positively correlated with the temperature, while *Methanothermobacter* was negatively correlated (*p* < 0.05). There were no archaeal taxa significantly correlated with ${\mathrm{SO}}_{4}^{2–}$, Mg^2+^, pH, oil viscosity, and mineralization.

## Discussion

### Functional Archaea in the Deep Subsurface Petroleum Reservoirs

Taxonomic distribution of archaeal communities in both aqueous and oil phases from four production wells was investigated. *Archaeoglobus* and *Methanothermobacter* represented as the most common microbes in each sample (**Figure 2A**). Other members (mostly were methanogens) with relative low abundance made up the remaining composition.

*Archaeoglobus* as hyperthermophilic sulfate-reducing archaea, its potential for anaerobic hydrocarbon oxidization has been reported (Stetter et al., 1993; Khelifi et al., 2010; Holmes et al., 2011). And confirmed by Khelifi et al. (2014) that *Archaeoglobus* (specifically, *A. fulgidus*) performed the anaerobic oxidation of long-chain *n*-alkanes (C_10_–C_21_) under sulfate-reducing conditions. *Geoglobus* and *Ferroglobus* appeared mainly in oil phase with low abundance are another two anaerobic hydrocarbon degrading microbes (**Figure 1B**). For *Ferroglobus, F. placidus* is the only isolated strain in this genus and it can grow by oxidizing benzoate and phenol to carbon dioxide with Fe(III) serving as the sole electron acceptor (Tor and Lovley, 2001; Holmes et al., 2011, 2012). Genus of *Geoglobus* contains two isolated members: *G. acetivorans* and *G. ahangari*. Genome analysis showed that *G. acetivorans* contains the ability of oxidizing aromatic compounds and *n*-alkanes through anaerobic respiration linked to Fe(III) reduction (Mardanov et al., 2015). Although *G. ahangari* does not contain any benzoate degradation genes (Manzella et al., 2015), it can completely oxidize long-chain fatty acids anaerobically (Zwolinski et al., 2000) together with other members of the *Archaeoglobales* (*F. placidus, G. acetivorans, A. fulgidus*, and others) (Klenk et al., 1997; Anderson et al., 2011; Mardanov et al., 2015). Fatty acids are not only the major components of crude oil (Atlas, 1981) but also the degradation by-products of complex organic matter and alkanes (Zwolinski et al., 2000; So et al., 2003). Besides, as another hyperthermophile suspected to anaerobically metabolize alkanes in the domain of archaea (Mardanov et al., 2009), *Thermococcus* consisted a very large proportion in the highly water flooded well of A1 sample. Thus, the appearances of these non-methanogen appeared as the dominant archaea (*Archaeoglobus* and *Thermococcus*) in the petroleum reservoir is not surprising. They may have played very important roles and performed the important processes like hydrocarbon degradation and fatty acids metabolism in the deep subsurface petroleum reservoir.

*Methanothermobacter* was the major division in all these samples especially in A1, A2, and A3 production wells. Actually, this type of methanogen was prevalent in many high-temperature reservoirs (Orphan et al., 2000, 2003; Bonch-Osmolovskaya et al., 2003; Nazina et al., 2006; Li et al., 2007, 2012; Mochimaru et al., 2007; Gray et al., 2009; Gieg et al., 2010; Mayumi et al., 2011; Mbadinga et al., 2012; Wang et al., 2012; Zhao et al., 2012). Genus of *Methanothermobacter* which was initially proposed by Boone in 1993 (Boone et al., 1993) is one kind of the hydrogenotrophic methanogens sharing the ability to produce methane from carbon dioxide (Wasserfallen et al., 2000). Some isolates are able to utilize formate for growth or as electron donor while sulfur as electron acceptor (Wasserfallen et al., 2000). Other methanogens in the water flooded petroleum reservoirs had different methanogenic pathways. *Methanosaeta* and *Methanosarcina* are aceticlastic methanogens, while *Methanosarcina* can utilize carbon dioxide and hydrogen to produce methane (Oren, 2014d). *Methanoculleus, Methanobacterium, Methanocalculus, Methanolinea*, and *Methanospirillum* as the methanogens generated methane through hydrogenotrophic methanogenesis or methylotrophic methanogenesis (Oren, 2014a,b,c,d,e). Methanol, methylated amines, dimethyl sulfide, or methanethiol can be used for methanogenesis by *Methanomethylovorans* (Oren, 2014d). *Methanomassiliicoccus* produce methane only by reducing methanol with hydrogen as the electron donor (Dridi et al., 2012). *Methermicoccus* as the first reported thermophilic methylotrophic methanogen can utilize methanol, methylamine, and trimethylamine to produce methane (Cheng et al., 2007; Oren, 2014f).

Since the process of methanogenic hydrocarbon degradation in the high-temperature petroleum reservoirs is considerably a complex process from the initial hydrocarbon substrates to the end-product methane, generally it involves various microorganisms including diverse taxonomy of bacteria and archaea. But as our investigation shows, besides a few suspected archaea with potential of anaerobic hydrocarbon oxidation, most of the rest archaea perform the downstream process of hydrocarbon degradation, like long chain and short chain fatty acids degradation or methanogenesis. Archaeal communities containing the potential of performing the degradation from hydrocarbon to the end-product methane could improve our understanding about archaea in this *in situ* subsurface ecosystem.

### Diversity of Archaeal Communities in Aqueous and Oil Phases

**FIGURE 4:**
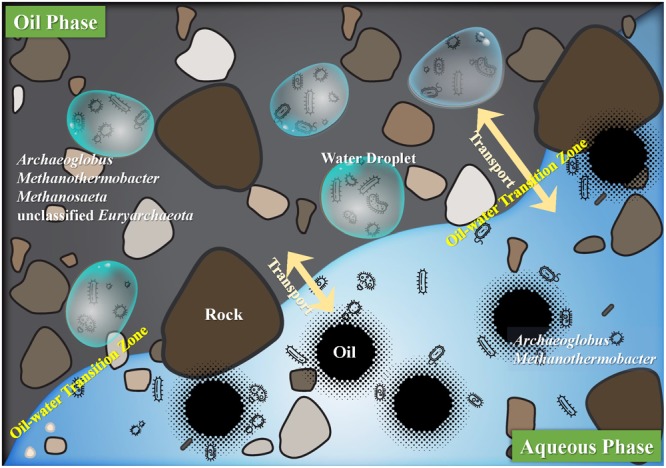
Hypothesized outline of oil and aqueous phases in the deep subsurface petroleum reservoirs. Interface of the oil and aqueous phases showing the oil–water transition zone where water droplets and oil droplets can transport to each other. And these droplets constitute the microscopic habitats where microorganisms interacted with the substrates. In the aqueous phase, microorganisms inhabit in the liquid water or around the oil droplets, and in the oil phase microorganisms inhabit only in the minuscule water droplets. Microorganisms showing in oil and aqueous phase represent the core archaea that appeared in the four production wells.

It is generally believed that the processes of anaerobic biodegradation of hydrocarbon is typically taking place at the oil–water interface (Head et al., 2003; Bennett et al., 2013). Recently, Meckenstock et al. (2014) showed that minuscule water droplets (1–3 μL) entrapped in oil are the places where microorganisms perform the metabolic activities. We hypothesized that in the aqueous and oil phases, the microscopic habitats are the places where microorganisms inhabit in. And the different microbial adhesion and functional properties in aqueous and oil phases constitute different types of microscopic habitats. In the petroleum reservoir, especially the oil–water transition zone, the water droplets and oil droplets can easily transport to each other. More specifically, in the aqueous phase, free microbes inhabit in the water and also around the minuscule oil droplets. But in the oil phase, all the microbes are supposed to be in the dispersed minuscule water droplets. In the microscopic habitats of aqueous and oil phases, the interface of oil and water droplets is the site where the biodegradation occurs (**Figure 4**).

Archaeal communities in different production wells cannot be seen as replicates, because of their independent ecological characteristics (**Table 1**). However, even when taking these production wells as replicates, oil and aqueous phases as treatment, the *p-*value of Choa1 boxplots was slightly >0.05 (Supplementary Figure S3). Moreover, both Chao1 and observed OTUs showed that in each independent production well, archaeal diversity was much higher in oil phase than that in the aqueous phase (Supplementary Figure S1). We conclude that this was an obvious difference between aqueous and oil phase in each independent production wells. To be more specific, microorganisms in the aqueous and oil phases of higher water cut production wells A1 and A2 (98.0 and 93.8% water cut) had the highest Bray–Curtis dissimilarity (0.95 and 0.77, respectively), while lower water cut production wells A3 and A4 (78.6 and 66.8% water cut) had the lowest Bray–Curtis dissimilarity (0.13 and 0.24, respectively). Bray–Curtis dissimilarity further statistically indicated the dissimilarity of archaeal composition in oil and aqueous phases from high water cut production wells were much higher than those from low water cut production wells (**Figure 2B**). All these results further confirmed that greater dissimilarity of the archaeal communities between the two phases appeared with the petroleum wells with higher water cut. However, it was just the opposite of the corresponding bacterial communities in this subsurface ecosystem: the greater bacterial diversity between the aqueous and oil phases appeared in the production fluids with lower water cut (Wang et al., 2014). Bacteria and archaea composed the main microorganisms of subsurface petroleum reservoirs, their unique morphological structures, functional features, and adhesion properties of hydrocarbon maybe the factors that shaped their distinct distribution in aqueous and oil phases.

Because of potential toxicity and high hydrophobicity, it was widely believed that oil was not a preferred habitat for microbes (Atlas and Atlas, 1991). But recent studies showed that various microorganisms have been detected in crude oil phase (Yoshida et al., 2005; Yamane et al., 2008; Wang et al., 2014; Cai et al., 2015). According to alpha-diversity analysis, both Chao1 and observed OTUs in oil phase were much higher than that in the aqueous phase (Supplementary Figure S1). These results suggested that biodiversity of archaea components in the oil phase was much higher than that in the aqueous phase. This conclusion is consistent with the corresponding bacterial biodiversity in the aqueous and oil phases: diversity of the bacterial community of the aqueous phase was lower than that of the oil phase (Wang et al., 2014). Moreover, PCA illustrated that all the four samples in oil phases were grouped together, whereas the samples in aqueous phases were diverged (**Figure 2A**). Archaeal communities in oil phase from different petroleum reservoirs tended to be more similar, but those in aqueous phase appeared to have more dissimilarity. This result was highly consistent with Venn diagram analysis that oil samples shared much more common OTUs (17 OTUs) than aqueous samples (3 OTUs) (Supplementary Figure S4). The common archaea appeared in all these high-temperature production wells were mainly hydrocarbon degraders and methanogens (*Archaeoglobus, Methanothermobacter, Methanosaeta*, and unclassified *Euryarchaeota*).

Whatever the source of water injection (seawater, river water, or even formation water) is, water flooding is regarded as a main contamination of oil reservoirs which may involve the exogenous microorganisms (Magot et al., 2000). However, how the introduction of bacteria via water flooding affects the subsurface ecosystem remains unknown (Struchtemeyer et al., 2011; Ren et al., 2015). Perhaps archaeal communities in the oil phases with less influenced by flooding with surface water were more closely related to the indigenous in the subsurface petroleum reservoirs, and the more pristine the communities the more similar they were. The unique microorganisms in the oil phase tend to have strong adhesion to the oil and the ability benefit their growth on and biodegradation of very poorly water-soluble hydrocarbon, such as *n*-alkanes and large polycyclic aromatic hydrocarbon that dissolved in a non-aqueous phase (Abbasnezhad et al., 2011). The inhabitants of microbes in the oil phases may experience a relatively stable habitat (microscopic habitats) to maintain the high phylogenetic diversity of archaea. Thus, in the microscopic habitats of oil phase, technically the dispersed minuscule water droplets in the oil could be a suitable habitat that containing the indigenous microorganisms which performing hydrocarbon metabolic activities. The differences of archaeal distribution in aqueous and oil phases can give a hint for the identification of indigenous and exogenous microorganisms in the subsurface petroleum reservoirs.

### Temperature as a Key Factor Affected Archaeal Diversity and Distribution

PerMANOVA analyses (Supplementary Table S1) showed that were the two factors that significantly affected archaeal distribution in the production wells. Other environmental factors would have a weaker influence on the spatial variation of archaeal communities according to production wells. The difference of the archaeal communities between oil and aqueous phases was more obvious in the petroleum wells with higher water cut. However, when taking microbial in the two phases of each production well as a whole, the water cut did not promote the significant differences of microbial communities among production wells. Microcosmically, microorganisms have their unique morphological structures, functional features, and adhesion properties of hydrocarbon and water, thus the water cut could affect the microbial distribution in aqueous and oil phase. All the production wells have been water flooded over 30 years, thus macroscopically, it could not significantly affect the overall microbial compositions from different petroleum ecosystems. In the high-temperature environments, however, temperature still greatly impacted on archaeal diversity and distribution (**Figure 3A**). Archaeal diversity in production wells was positively correlated with temperature (55–65°C). Specifically, in the range of 55–65°C, *Archaeoglobus, Geoglobus*, and unclassified *Archaeoglobaceae* were positively correlated with the temperature while *Methanothermobacter* was the opposite (**Figure 3B**). One of the reason could be related to their temperature tolerance that *Archaeoglobaceae* thrive at temperature between 70 and 80°C (Brileya and Reysenbach, 2014) which is much higher than *Archaeoglobaceae* between 55 and 70°C (Oren, 2014a). In this high-temperature petroleum reservoir, temperature as an environmental factor may be beneficial to preserve microbial diversity especially those with high-temperature tolerance.

## Author Contributions

L-YW, J-DG, and B-ZM conceived the study. BL, L-YW, and KZ performed the experiments and data analysis. BL drafted the manuscript. J-FL and S-ZY collected the experimental samples from Shengli oilfield. J-DG and B-ZM were involved in the revision of the manuscript. All authors approved the final manuscript.

## Conflict of Interest Statement

The authors declare that the research was conducted in the absence of any commercial or financial relationships that could be construed as a potential conflict of interest.
