# Supplementary material for: Different Diversity and Distribution of Archaeal Community in the Aqueous and Oil Phases of Production Fluid From High-Temperature Petroleum Reservoirs
Source: Front Microbiol. 2018 Apr 27;9:841. doi: 10.3389/fmicb.2018.00841 (PMC5934436; doi:10.3389/fmicb.2018.00841)
Supplement: Supplementary file 1 [file Data_Sheet_1.pdf]

Supplementary materials

Insight into archaeal communities in the aqueous and oil phases of production water from a high-temperature petroleum reservoir

Bo Liang[1], Kai Zhang[1], Li-Ying Wang[1], Jin-Feng Liu[1, 3], Shi-Zhong Yang[1, 3], Ji-Dong Gu[2], Bo-Zhong Mu[1, 3, *]

[1]State Key Laboratory of Bioreactor Engineering and Institute of Applied Chemistry, East China University of Science and Technology, Shanghai, P.R. China

[2]School of Biological Sciences, The University of Hong Kong, Pokfulam Road, Hong Kong, P.R. China

[3]Shanghai Collaborative Innovation Center for Biomanufacturing Technology, Shanghai 200237, P.R. China

*Correspondence: Bo-Zhong Mu

E-mail: bzmu@ecust.edu.cn

Phone: +86 21 64252063; Fax: +86 21 64252485

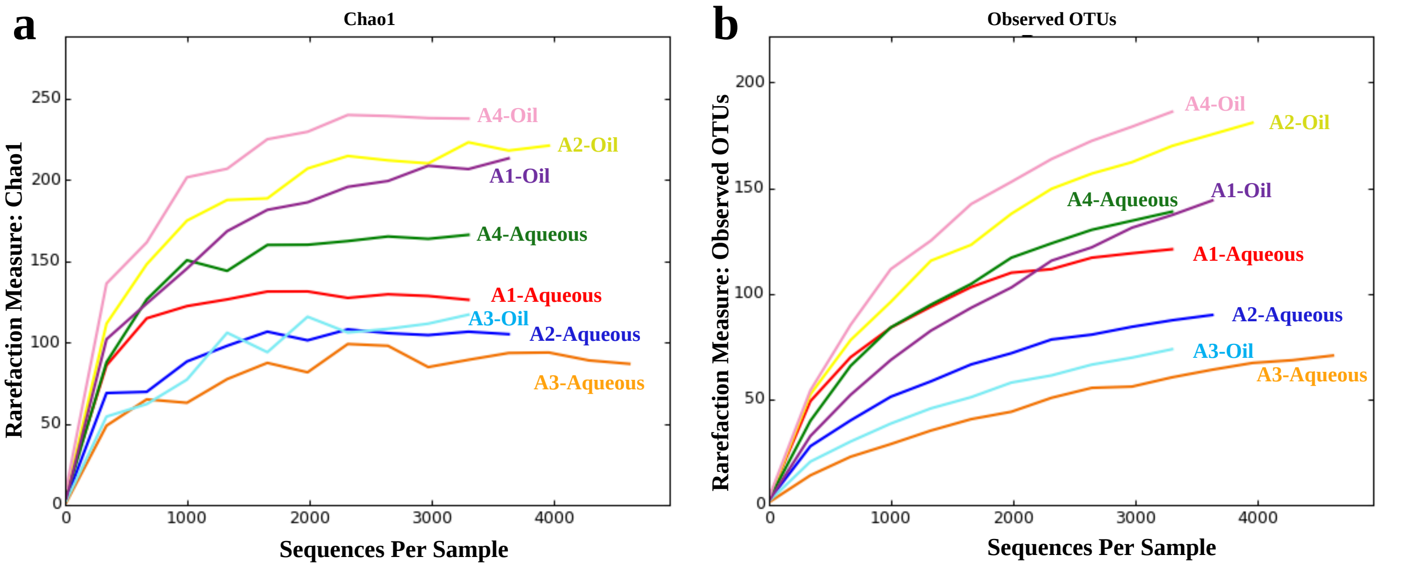

Figure S1. OTU numbers based multi-samples rarefaction curves (a) and Shannon curves (b) at 97 % similarity.

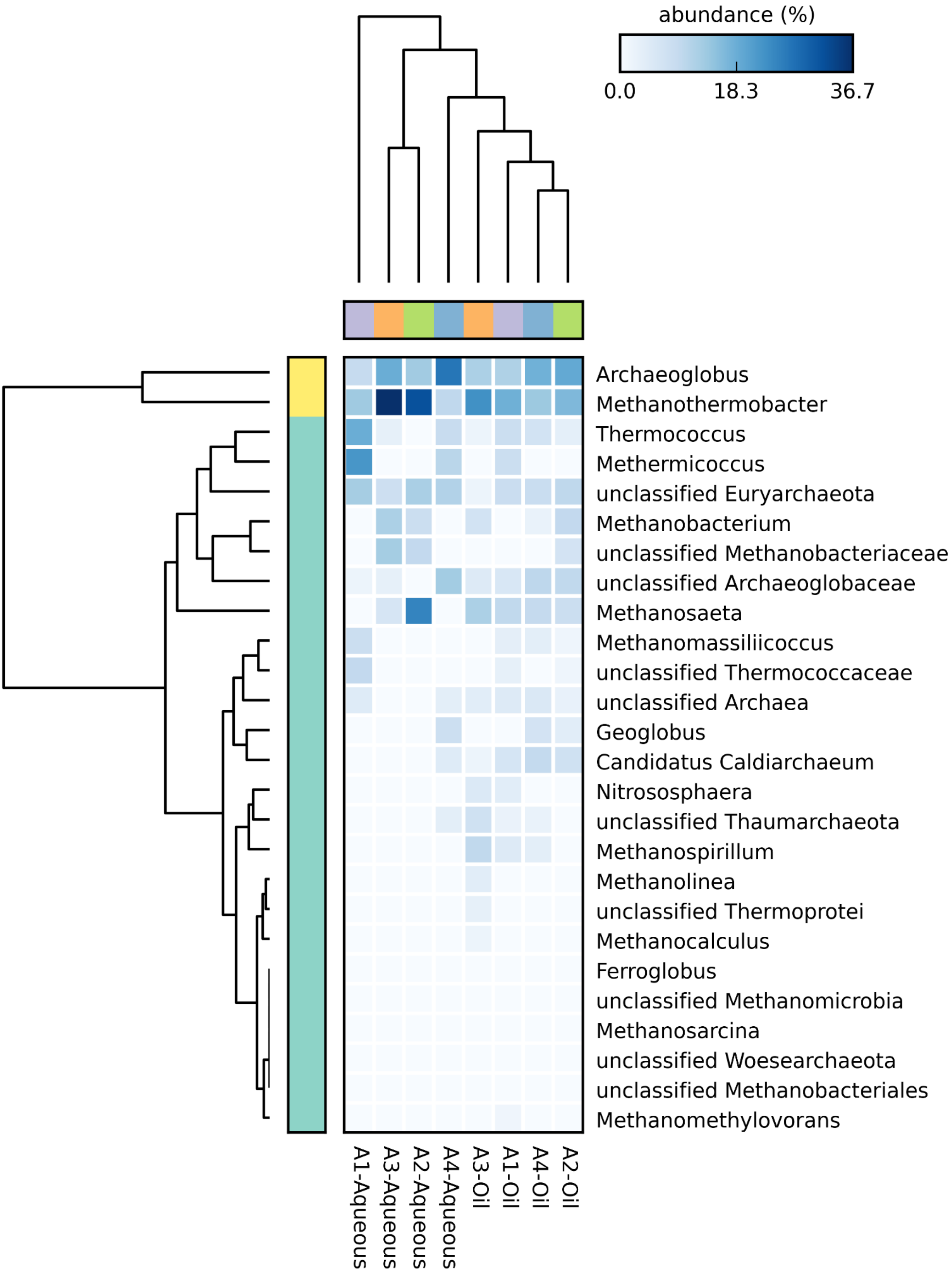

Figure S2. Log-normalized heat map, hierarchical clustering of samples using genus-level distributions based on phylogenetic analysis of 16S rRNA gene pyrosequencing sequences. The log-normalized heat map shows the relative percentage of each archaea genus (variables clustering on the vertical axis) within each sample (horizontal axis clustering). The depicted color key indicates the log-transformed values of abundance percentage values in each sample. The samples label with "A1-Aqueous", "A2-Aqueous", "A3-Aqueous", "A4-Aqueous", "A1-Oil", "A2-Oil", "A3-Oil" and "A4-Oil" representing samples in aqueous and oil phases of the four production wells (C7-J9, C6-15, C6-G10 and CN13-13) from Shengli oil field

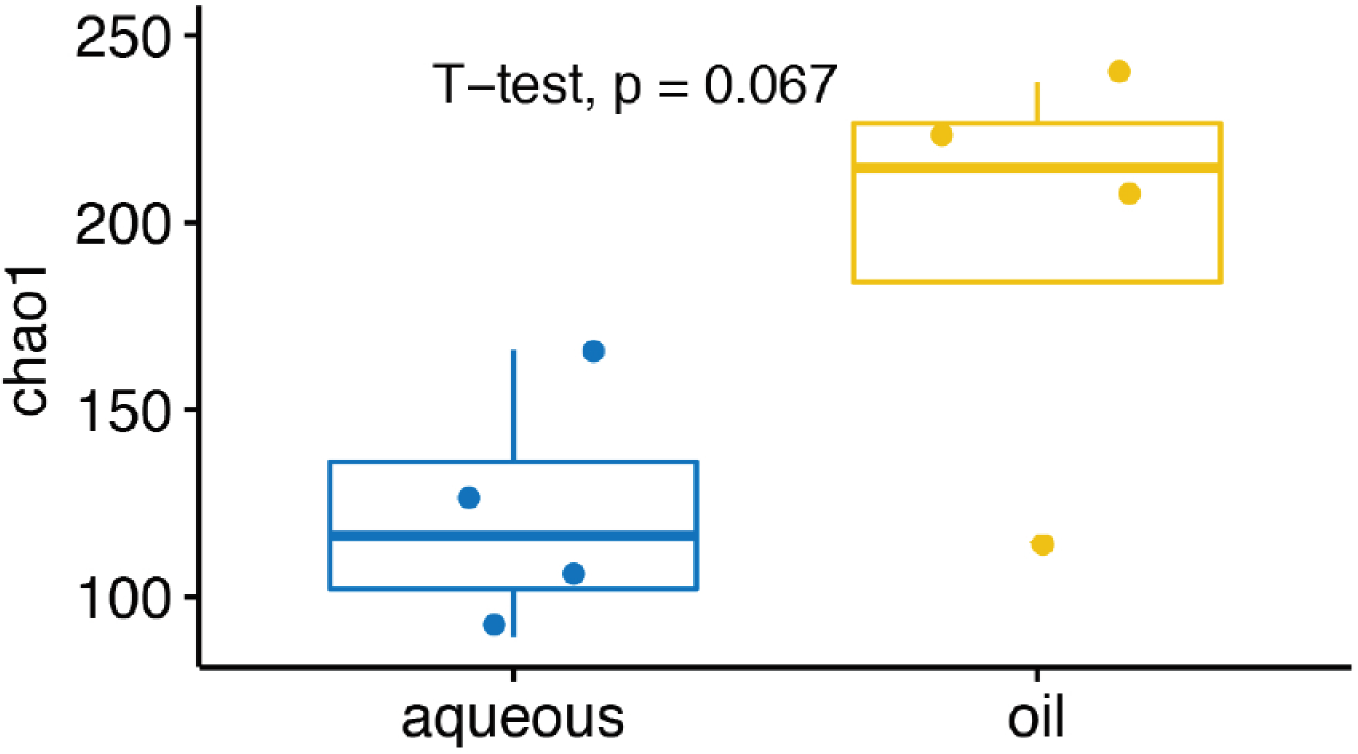

Figure S3 . Boxplots showing Choa1 between oil and aqueous samples measured by Student T-Test (*p* = 0.067). Each blue and yellow point represents one sample in the aqueous and oil phase.

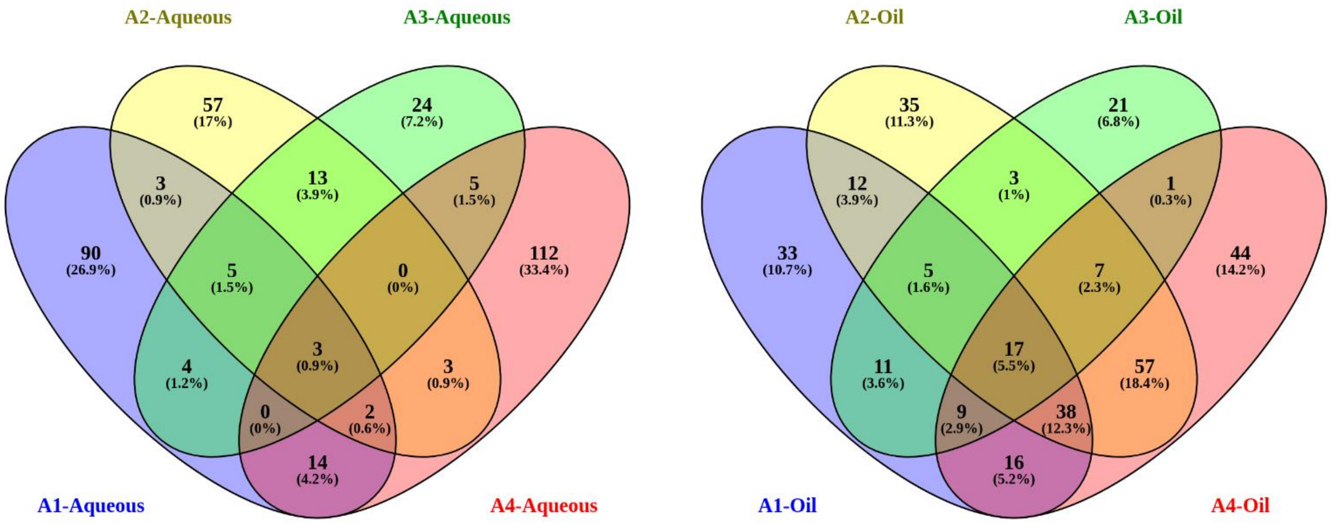

Figure S4. Venn diagram showing the unique and shared OTUs (3% distance level). (a) OTU distribution of A1-Aqueous, A2-Aqueous, A3-Aqueous, A4-Aqueous samples of four production wells. (b) OTU distribution of A1-Oil, A2-Oil, A3-Oil and A4-Oil samples of four production wells.

Table1 . Significant effect of environmental variables on the archaeal composition in the different production wells based on the rarefied OTUs at the same sequencing depth (3300 reads) using PerMANOVA analyses (Adonis function, 999 permutations).

	R^2	P value
Water cut (%)	0.18236	0.281
Cl^- (mg L^{-1})	0.45268	0.02
SO_4^{2-} (mg L^{-1})	0.18585	0.381
K^+ and Na^+ (mg L^{-1})	0.3245	0.071
Ca^{2+} (mg L^{-1})	0.12611	0.556
Mg^{2+} (mg L^{-1})	0.20826	0.231
pH	0.06491	0.788
Oil viscosity (mPa.s)	0.1514	0.339
Temperature (° C)	0.18236	0.281
Mineralization (mg L^{-1})	0.45268	0.02
